# Divergent Adaptive Strategies by Two Co-occurring Epiphytic Orchids to Water Stress: Escape or Avoidance?

**DOI:** 10.3389/fpls.2016.00588

**Published:** 2016-05-03

**Authors:** Wei Zhang, Hong Hu, Shi-Bao Zhang

**Affiliations:** ^1^Key Laboratory of Economic Plants and Biotechnology, Kunming Institute of Botany, Chinese Academy of SciencesKunming, China; ^2^Yunnan Key Laboratory for Wild Plant ResourcesKunming, China; ^3^University of Chinese Academy of SciencesBeijing, China

**Keywords:** epiphyte, drought, coexistence, adaptive strategy, water relations, photosynthesis, leaf phenology, functional trait

## Abstract

Due to the fluctuating water availability in the arboreal habitat, epiphytic plants are considered vulnerable to climate change and anthropogenic disturbances. Although co-occurring taxa have been observed divergent adaptive performances in response to drought, the underlying physiological and morphological mechanisms by which epiphyte species cope with water stress remain poorly understood. In the present study, two co-occurring epiphytic orchids with different phenologies were selected to investigate their drought-resistance performances. We compared their functional traits, and monitored their physiological performances in a 25-days of drought treatment. In contrast to the deciduous species *Pleione albiflora*, the evergreen species *Coelogyne corymbosa* had different root anatomical structures and higher values for saturated water content of pseudobulbs. Moreover, plants of *C. corymbosa* had thicker leaves and epidermis, denser veins and stomata, and higher values for leaf mass per unit area and the time required to dry saturated leaves to 70% relative water content. However, samples from that species had lower values for net photosynthetic rate (*A*_n_), stomatal length and chlorophyll content per unit dry mass. Nevertheless, due to greater capacity for water storage and conservation, *C. corymbosa* maintained higher *A*_n_, stomatal conductance (*g*_s_), and instantaneous water-use efficiency during severe drought period, and their values for leaf water potential were higher after the water stress treatment. By Day 10 after irrigation was restarted, only *C. corymbosa* plants recovered their values for *A*_n_ and *g*_s_ to levels close to those calculated prior to the imposition of water stress. Our results suggest that the different performance responding to drought and re-watering in two co-occurring epiphytic orchids is related to water-related traits and these two species have divergent adaptive mechanisms. Overall, *C. corymbosa* demonstrates drought avoidance by enhancing water uptake and storage, and by reducing water losses while *P. albiflora* employs a drought escape strategy by fixing more carbon during growing season and shedding leaves and roots at dry season, leaving a dormant pseudobulb to minimize transpiration. These findings may improve our understanding of the potential effects that climate change can have on the population dynamics of different epiphytic taxa.

## Introduction

Vascular epiphytes use other plants as a support for growth but do not parasitize hosts (Benzing, 1990). This taxonomically heterogeneous group, comprising over 27,000 species in 73 families, accounts for approximately 9% of all extant vascular plant species (Zotz, 2013). Vascular epiphytes are most impressive in tropical rainforests, and their abundance and diversity are noticeably decreased outside the tropic zone. Some noteworthy exceptions include temperate rain forests of Chile and New Zealand, or montane forests in the Himalayas, where the epiphyte biomass and diversity are comparable to that of many tropical forests (Zotz, 2005).

Epiphytic plants represent a substantial proportion of the entire biomass in tropical montane cloud forests (Hofstede et al., 1993; Nadkarni et al., 2004) and temperate rain forests (Diaz et al., 2010). Both epiphytes and the humus they generate and hold in the canopy can influence the processes of water/nutrient uptake or release. Therefore this plant group plays a key role in hydrology and nutrient cycling within forest ecosystems (Veneklaas et al., 1990; Hofstede et al., 1993; Nadkarni et al., 2004; Pypker et al., 2006; Diaz et al., 2010). In addition, epiphytes facilitate animal life in tree canopies by providing abundant and diverse resources, e.g., food and shelters (Treseder et al., 1995; Bluthgen et al., 2000; Ellwood et al., 2002).

Although epiphytes are essential components of these ecosystems, this plant group is currently considered more vulnerable to global climate change and anthropogenic disturbances when compared with co-occurring plant types such as supporting trees (Benzing, 1998; Zotz and Bader, 2009; Mondragón et al., 2015). This is probably because arboreal habitats cause epiphytes tightly coupled to atmospheric inputs whereas terrestrial plants are in contact with the soil, from which they can obtain stable supplies of water and nutrient (Benzing, 1998). Among all the abiotic factors, water appears to be one of the greatest limitations to the growth of vascular epiphytes among all the abiotic factors (Laube and Zotz, 2003; Zotz et al., 2010). Furthermore, the diversity and abundance of epiphytic species respond more emphatically to water gradients than any other life forms, including trees, shrubs, and terrestrial herbs (Gentry and Dodson, 1987; Benzing, 1990). The high mortality rate for juvenile epiphytic plants is probably caused by drought or low air humidity (Zotz and Hietz, 2001). Unlike a terrestrial habitat, the supply of moisture in a tree canopy is intermittent rather than continuous. Rainless periods of a few hours may be sufficient to induce drought stress (Zotz and Hietz, 2001). However, even in the wet season, rainless periods of a few days are rather common (Zotz and Tyree, 1996; Engelbrecht et al., 2006). Epiphytic species endure at least moderate drought stress over most of the year (Zotz and Tyree, 1996). Therefore, multiple morphological and physiological adaptations have evolved to cope with the challenges of a fluctuating external water supply. Examples include water-impounding foliage of bromeliads (Zotz and Thomas, 1999), succulent pseudobulbs produced by orchids (Ng and Hew, 2000), and the water-saving crassulacean acid metabolism, or CAM (Zotz and Hietz, 2001; Lüttge, 2004). However, when one considers how the frequency of drought events is increasing in certain regions (IPCC, 2013), along with more variable precipitation patterns (Allan and Soden, 2008) and rapid deforestation/fragmentation in both tropical and temperate forests (Echeverria et al., 2006; Broadbent et al., 2008), one must ask whether these adaptions are enough to support the survival of epiphytes in our changing world.

The answer seems ambiguous. For example, epiphyte species richness is much lower in a secondary forest than in an adjacent primary forest, and the number of species within some taxa declined dramatically while the tallies of others remained flat (Krömer and Gradstein, 2003). Some taxa have benefited from anthropogenic disturbances even if the entire epiphytic species diversity has been dramatically affected (Barthlott et al., 2001; Cascante-Marin et al., 2006; Toledo-Aceves et al., 2012). Epiphytic taxa exhibit divergent patterns of growth, survival, and the establishment of new seedlings on isolated remnant trees after they are exposed to a shift in microclimate due to logging, with mesic taxa tending to be more severely affected and substituted by more xerophytic ones (Padmawathe et al., 2004; Werner and Gradstein, 2008; Werner, 2011). These reports indicate that various epiphytic species differed in their responses to environmental changes, with even slight shifts in their microclimate producing “winners” and “losers” (Zotz and Bader, 2009). However, it is unclear whether these differences in sensitivity are related to the way in which they adapt to water stress.

Three commonly recognized adaptive strategies for a plant to water stress are drought escape, drought avoidance, and drought tolerance (Levitt, 1980; Farooq et al., 2012). Drought escape means that a plant to completes its life cycle and goes dormant before the onset of the dry season. Plants that utilize drought avoidance maintain a high water status by enhancing their water uptake and reducing water losses. By contrast, drought-tolerant plants continue their metabolic activities even at low tissue water potential. The boundaries between types of strategies are not clear-cut because they are not mutually exclusive (Levitt, 1980). Plants with different drought adaptive strategies may coexist within the same habitat, especially in some arid and semi-arid ecosystems (Chaves et al., 2002; Galmés et al., 2007; Hernández et al., 2010).

Although these divergent performances have been widely reported among co-occurring epiphytic taxa (Padmawathe et al., 2004; Werner and Gradstein, 2008; Werner, 2011; Toledo-Aceves et al., 2012), little is known about the physiological and morphological mechanisms behind this phenomenon. With almost 19,000 epiphytic species, the family Orchidaceae alone accounts for 68% of all epiphytes (Zotz, 2013). This family displays a spectacular array of adaptations and rapid speciations with numerous adaptive characteristics (Silvera et al., 2009). For this present study, we compared two co-occurring epiphytic orchids with different phenologies: the evergreen *Coelogyne corymbosa* and deciduous *Pleione albiflora*. Their functional traits (including those of the root, pseudobulb, and leaf) were compared with regard to plant water relations, and their physiological performances were monitored over 25 days of drought treatment, followed by 10-days of recovery after re- irrigation. The main aims were to explore their respective adaptive strategies and investigate any possible links with performance in response to drought conditions. Our goal was to improve our understanding of the population dynamics of different types of epiphytes in a changing world so that we can preserve this vulnerable group. We tested three hypotheses:

(1) With thick, evergreen leaves, plants of *C*. *corymbosa* employ a drought avoidance strategy while the deciduous *P*. *albiflora* escape the effects of drought by shedding its leaves and roots during the dry season.
(1) Plants of *C. corymbosa* are better able to resist drought while *P*. *albiflora* shows weaker capacity for drought-resistance but stronger photosynthetic performance during favorable growing periods.
(1) Because of their enhanced capacity for water conservation, epiphytic species that utilize a drought avoidance strategy will be the “winners” if their local environment becomes drier or an extreme drought event occurs.

## Materials and Methods

### Plant Materials and Growth Conditions

Although *C. corymbosa* and *P. albiflora* are both perennial herbs they have distinct morphologies and phenologies. While the former has two evergreen leaves on each perennial pseudobulb, the latter features only one leaf on its annual renewed pseudobulb. According to their carbon isotope ratios (-28.23% for *C*. *corymbosa* and -29.47% for *P*. *albiflora*), leaves of both species use C_3_ photosynthetic CO_2_ fixation. We did not test the photosynthetic modes of green pseudobulbs in these two species, but CAM photosynthetic pathway may be presented in green pseudobulbs of some orchids (Ng and Hew, 2000). When the growth of *P. albiflora* begins in the spring, fresh roots are produced from the base of the new shoot at the side of the pseudobulb. The new shoot swells and the old pseudobulb shrinks during the growth of the plant. As winter approaches, the single leaf withers, the old pseudobulb shrivels, and the roots die, leaving the new pseudobulb to survive the seasonal cold and dry conditions (Cribb and Butterfield, 1999). Contrary to the annual renewed nature of pseudobulb and leaf in *P. albiflora*, Plants of *C. corymbosa* persist leaves and pseudobulbs for years (even the leaves dropped, the leafless pseudobulbs survive) and lack a dormant period (Cribb and Butterfield, 1999). Plants of *C. corymbosa* are wide distributed from Nepal to southwest China while those of *P. albiflora* are relatively limited to western portions of Yunnan Province in southwest China and adjacent northern Myanmar (Chen et al., 2009). The two orchids examined here were found on the trunks of *Illicium* sp., *Lithocarpus* sp., and *Rhododendron delavayi* trees in an evergreen broad-leaved forest in the Xiaoheishan Nature Reserve in Yunnan Province, at the elevation of 2500 m. Samples of each were collected in April 2014, at the end of the dry season, when plants of *P. albiflora* were still in dormancy (leafless and rootless) and the new shoots of *C. corymbosa* were also dormant.

The plants were grown in a greenhouse at Kunming Institute of Botany, CAS (1990 m, E102°41′, N25°01′) in baskets (30 cm × 40 cm) containing a mixture of 70% bark (1 cm × 1 cm), 20% moss, and 10% humus. The light intensity was controlled with shade nets to provide a level approximating 40% of full sunlight (800 μmol m^-2^ s^-1^). A humidification and ventilation system was used to maintain the ambient temperature at 20 to 30°C and the relative humidity above 60% throughout the growing season. Before the treatment began, plants were watered normally at 3-days intervals. Plants grew in the greenhouse for 3 months, and the current year’s fully expanded leaves from each species were used in our analysis.

### Functional Traits of Leaf, Pseudobulb, and Root

Six leaves and newly mature pseudobulbs were sampled from different plants, and immediately weighed to obtain their fresh weight (FW). Leaf area were measured with a leaf area meter (3100, Li-Cor, USA). Afterward, those materials with cutting edges were saturated overnight in distilled water before recording values for saturated weight (SW). The samples were then oven-dried at 80°C for 48 h before determining dry weight (DW). Relative water content (RWC) was calculated as (FW-DW)/(SW-DW) × 100. The saturated water content (SWC) of the pseudobulbs, an indicator of water storage capacity, was computed as (SW-DW)/DW (Stratton et al., 2000). Specific leaf weight was expressed as leaf dry mass per unit area (LMA). Chlorophyll was extracted in 10 mL of N, *N*-dimethylformamide in volumetric flasks for 48 h under darkness. Absorbance was measured at 647 and 664 nm with a spectrophotometer (UV-2550; Shimadzu, Japan) to calculate total chlorophyll content, following the method of Inskeep and Bloom (1985).

The rate of water loss after excision was monitored from the undamaged, mature leaves that were first saturated overnight to obtain SW values. The leaves were then placed on a lab bench and their cut petioles were sealed with Parafilm. FWs were measured periodically for 10 days on a digital balance before the leaves were oven-dried at 80°C for 48 h to obtain DW. As a threshold for physiological damage, the time needed to dry saturated leaves to 70% RWC (*T*_70_) was determined by regressing RWC against the time interval from leaf excision to each measurement of FW (Hao et al., 2010).

For each species, the middle portions of their sampled leaves, pseudobulbs, and maturation zone of roots were first fixed in FAA solution (formalin, acetic acid, ethanol, and distilled water; 10:5:50:35, v:v:v:v) for a month before transverse sections of each organ were made with a cryostat microtome (CM3050S; Leica, Germany). They were then photographed under a light microscope (DM2500; Leica, Germany). Leaf traits of thickness (LT), upper cuticle thickness (UCT), upper epidermal thickness (UET), lower epidermal thickness (LET), and mesophyll thickness (MT) were then measured from those digital photographs with the Image J software (National Institutes of Health, USA).

The adaxial and abaxial epidermises were peeled from the leaves, and images were made under the DM2500 light microscope. Stomatal parameters were measured from those digital photographs with the Image J software. For each leaf, stomata were tallied in six randomly selected fields. Stomatal density (SD) was calculated as the number per unit leaf area. Stomatal length (SL) was measured from 20 stomata selected randomly. For determining vein density (VD), leaves were boiled for 5 min in a 1% NaOH solution and rinsed in distilled water before being photographed. Total vein length was measured by Image J software and VD was calculated as total vein length per area. For each species, six leaves from different plants were used for anatomical observations.

### Drought and Re-watering Experiments

During July and August of 2014, 25-days drought and 10-days re-watering experiments were performed. The net photosynthetic rate (*A*_n_) and maximal photochemical efficiency of Photosystem II (*F*_v_/*F*_m_) of the leaves as well as the water potential of the culture substrate (Ψ_cs_), were measured at 5-days intervals during the drought period and on Days 1, 3, and 10 after normal irrigation resumed. Pre-dawn water potential of leaves (Ψ_leaf_) from each species was measured at the beginning and the end of the drought period. A group of well-watered plants was set as a control to compare the lifespan of leaves.

Values for *A*_n_ and *F*_v_/*F*_m_ were obtained with a portable photosynthesis system equipped with a fluorescence chamber (6400-40; Li-Cor, USA). While the photosynthetic measurements were being made, the light level was set at a saturation intensity of 600 μmol m^-2^ s^-1^ (based on photosynthetic light response curves, data not shown) and the atmospheric CO_2_ concentration was kept at 400 μmol mol^-1^ by a CO_2_ injector system (6400-01; Li-Cor, USA). Measurements of *A*_n_ were recorded from 09:00 to 11:30, when CO_2_ uptake was maximal. Values for stomatal conductance (*g*_s_) and transpiration rate (*E*) were obtained simultaneously during the measurements of *A*_n_. Instantaneous water use efficiency (WUE_i_) was expressed as *A*_n_/*E*. Measurements of *F*_v_/*F*_m_ were conducted at night after the leaves had adapted to darkness for at least 3 h. Both Ψ_cs_ and Ψ_leaf_ were determined with WP4C Dew point PotentiaMeter (Decagon, USA). Five to six leaves from different plants were used in the drought and re-watering experiments.

### Data Analysis

Differences in leaf traits between species were assessed by independent *t*-tests. One-way ANOVA was used to test for significant differences before and after drought stress for both species, with means discriminated using Tukey’s multiple comparison test. The results were accepted as significant at *p* < 0.05. All statistical analyses were conducted with the SPSS 16.0 program (SPSS Inc., USA).

## Results

**FIGURE 1:**
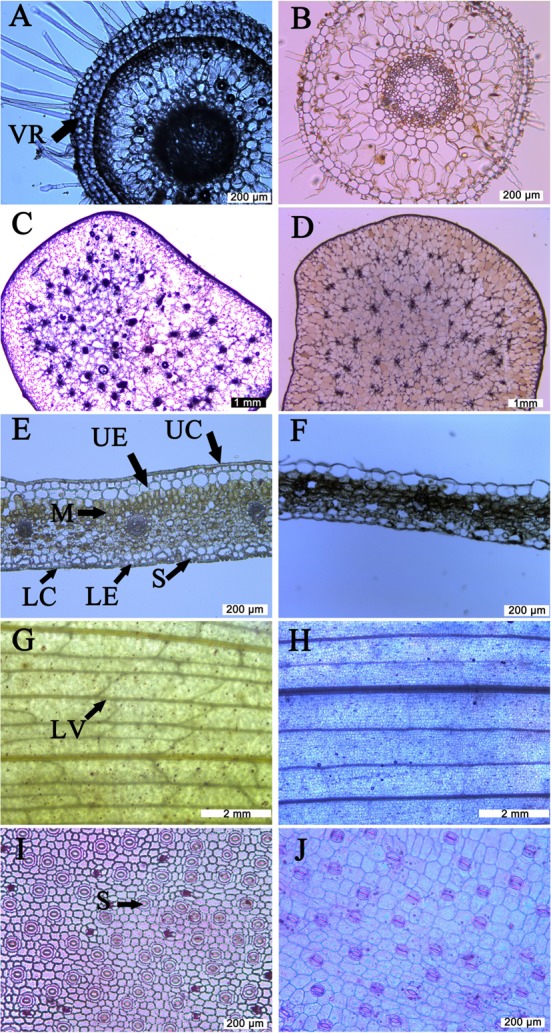
**Anatomic observation of different organs from *Coelogyne corymbosa* and *Pleione albiflora*.** **(A)** Root cross section from *C. corymbosa*; **(B)** root cross section from *P. albiflora*; **(C)** pseudobulb cross section from *C. corymbosa*; **(D)** pseudobulb cross section from *P. albiflora*; **(E)** leaf cross section from *C. corymbosa*; **(F)** leaf cross section from *P. albiflora*; **(G)** leaf veins from *C. corymbosa*; **(H)** leaf veins from *P. albiflora*; **(I)** lower epidermis from leaf of *C. corymbosa*; **(J)** lower epidermis from leaf of *P. albiflora*. VR, velamen radicum; UC, upper cuticle; UE, upper epidermis; M, mesophyll; LC, lower cuticle; LE, lower epidermis; S, stomata; LV, leaf vein.

Root cross-sections are shown in **Figures 1A,B**. Those of *C. corymbosa* were covered with a velamen consisting of four layers of dead cells while the epidermis of *P. albiflora* roots had a single cell layer. Structural cross-section indicated that velamen radicum was present for *C. corymbosa* but absence for *P. albiflora*. Roots of the former also exhibited a suberized exodermis.

The pseudobulbs from both species were anatomically similar, comprising a thick epidermis, multiple parenchyma cells, and vascular bundles (**Figures 1C,D**). Although there was no significant difference in FW (data not shown), *C. corymbosa* had a significantly higher SWC of pseudobulb (**Table 1**), indicating the pseudobulb of the former had a greater capacity for water storage.

**Table 1:** Functional traits of leaves and pesudobulbs from *Coelogyne corymbosa* and *Pleione albiflora*.

Trait	Function	*C. corymbosa*	*P. albiflora*	Significance
SWC (g g^-1^)	Water storage	8.36 ± 0.39	5.67 ± 0.43	0.001^∗∗∗^
LMA (g m^-2^)	Water availability and energy exchange	107.62 ± 6.08	41.32 ± 4.10	0.000^∗∗∗^
UET (μm)	Water conservation	76.84 ± 3.32	50.87 ± 3.69	0.000^∗∗∗^
UCT (μm)	Water conservation	11.86 ± 0.71	11.88 ± 0.47	0.983^ns^
LET (μm)	Water conservation	68.75 ± 2.50	40.55 ± 1.75	0.000^∗∗∗^
LCT (μm)	Water conservation	9.69 ± 1.13	9.03 ± 0.33	0.611^ns^
MT (μm)	Photosynthesis	242.69 ± 16.19	219.42 ± 8.31	0.222^ns^
LT (μm)	Water availability	391.12 ± 17.93	303.10 ± 9.10	0.001^∗∗∗^
MT/LT	Photosynthesis	0.62 ± 0.02	0.72 ± 0.02	0.000^∗∗∗^
VD (mm mm^-2^)	Water transport	1.86 ± 0.04	1.32 ± 0.06	0.000^∗∗∗^
SL (μm)	Gas exchange	38.26 ± 1.15	58.99 ± 0.84	0.000^∗∗∗^
SD (mm^-2^)	Gas exchange	74.93 ± 3.78	28.78 ± 2.93	0.000^∗∗∗^
*T*_70_ (h)	Water loss	166.65 ± 26.04	43.86 ± 5.94	0.001^∗∗∗^
CCA (μg cm^-2^)	Photosynthesis	52.8 ± 2.67	47.05 ± 1.87	0.116^ns^
CCM (mg g^-1^)	Photosynthesis	4.90 ± 0.25	11.38 ± 0.45	0.000^∗∗∗^


*SWC, saturated water content of pseudobulb; LMA, leaf mass per unit area; UET, upper epidermal thickness; UCT, upper cuticle thickness LET, lower epidermal thickness; LCT, lower cuticle thickness; MT, mesophyll thickness; LT, leaf thickness; VD, vein density; SL, stomatal length; SD, stomatal density; *T*_70_, time required for drying of saturated leaves to 70% RWC; CCA, chlorophyll content per unit area; CCM, chlorophyll content per unit dry mass. Data are means ± SE (*n* = 6–8). ns, *p* > 0.05; ^∗∗∗^*p* < 0.001.*

**FIGURE 2:**
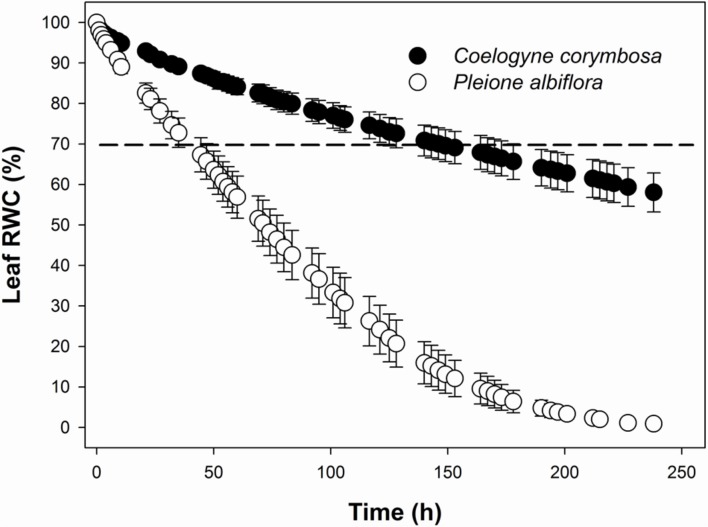
**Water loss curves for leaves from *C. corymbosa* and *P. albiflora*, showing changes in relative water content (RWC) with time after excision.** Dashed line indicates time required for drying of saturated leaves to 70% RWC (*T*_70_). Data are means ± SE (*n* = 6).

Most leaf traits also differed significantly between species. Compared with *P. albiflora*, leaves of *C. corymbosa* were thicker and had denser veins as well as denser but smaller stomata (**Figures 1E–J**; **Table 1**). These variations were manifested by their higher values for LMA, UET, LET, LT, VD, SD, and *T*_70_, but lower values for SL, and CCM (**Figure 2**; **Table 1**). However, values calculated for UCT, LCT, MT, and CCA did not differ significantly between species (**Table 1**). Thus, the thicker leaf of *C. corymbosa* was mainly caused by its thicker epidermis.

**FIGURE 3:**
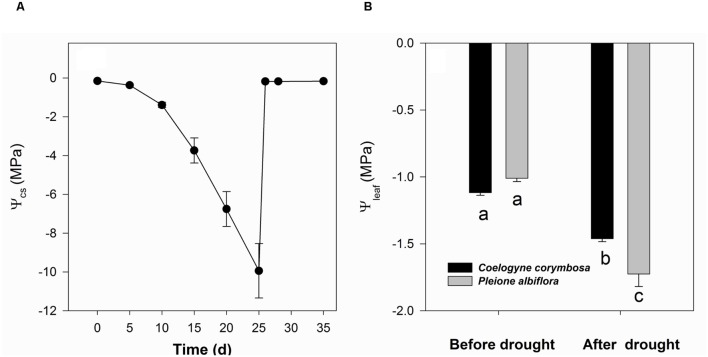
**Water potential in culture substrate (Ψ_cs_; **A)** and pre-dawn water potential of leaves (Ψ_leaf_; **B**) from *C. corymbosa* and *P. albiflora*.** Different letters indicate significant differences at *p* < 0.05 between species and treatments. Data are means ± SE (*n* = 5–6).

From the beginning of the drought treatment, Ψ_cs_ decreased continuously and dropped dramatically after 10 days of imposed stress. However, those values quickly rebounded to a very high level when the plants were re-watered (**Figure 3A**). Drought conditions decreased the pre-dawn Ψ_leaf_ readings for both species. Although *P. albiflora* showed comparable Ψ_leaf_ to *C. corymbosa* before irrigation was initially withheld, that species had significantly lower values after 25 days of drought stress (**Figure 3B**).

**FIGURE 4:**
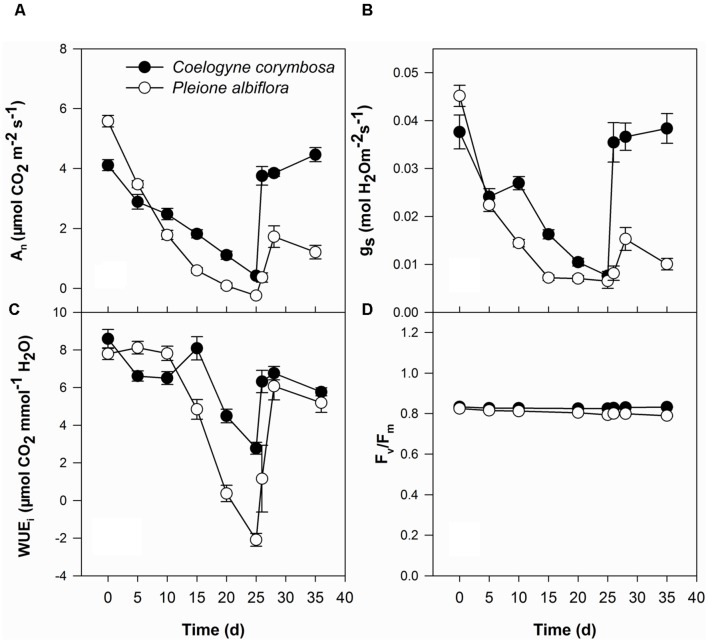
**Net photosynthetic rate (*A*_n_; **A)**, stomatal conductance (*g*_s_; **B)**, instantaneous water use efficiency (WUE_i_; **C)**, and maximal photochemical efficiency of photosystem II (*F*_v_/*F*_m_; **D)** for *C. corymbosa* and *P. albiflora* under drought treatment (Days 0–25) and after re-watering (Days 26–35).** Data are means ± SE (*n* = 6).

Under well-watered conditions, values of *A*_n_ were significantly higher in *P. albiflora* than in *C. corymbosa* (**Figure 4A**). For both species, *A*_n_ and *g*_s_ decreased as drought stress intensified although those values were significantly higher in *C. corymbosa* than in *P. albiflora* after 15 days of drought treatment (**Figures 4A,B**). The WUE_i_ in *P. albiflora* was high under well-watered conditions and moderate drought (i.e., Days 5 and 10 after irrigation was stopped), but was decreased dramatically under severe drought (after 15 days of treatment). By contrast, *C. corymbosa* showed relatively higher WUE_i_ under severe drought (**Figure 4C**). After 25 days of treatments, *F*_v_/*F*_m_ values were significantly decreased in both species. However, all *F*_v_/*F*_m_ values were relatively stable (close to 0.8) under drought stress (**Figure 4D**). When the plants were re-watered, values of *A*_n_, *g*_s_, WUE_i_, and *F*_v_/*F*_m_ increased in both species. After 10 days of recovery, levels of these photosynthesis representing parameters in *C. corymbosa* were nearly as high as those measured before the drought treatment. However, *P. albiflora* were still significantly lower than those calculated prior to the imposition of drought. Meanwhile, when compared with leaves from well-watered plants, 25 days of water stress significantly decreased the lifespan of leaves in *P. albiflora* (121 ± 3.7 days vs. 160 ± 2.8 days; *n* = 6).

## Discussion

Our study revealed that these two co-occurring epiphytic orchids differ significantly in morphological and physiological traits related to the uptake, storage, transport, and loss of water. Furthermore, the photosynthetic performance during drought treatment and recovery differed significantly between them. These results indicate that the two species have divergent adaptive strategies for coping with drought stress.

Velamen radicum and suberized exodermis were present in *C. corymbosa* but absence in *P. albiflora*. The velamen radicum is an important adaptive structure for orchids in epiphytic habitats because it allows for rapid water-absorption under wet conditions but also provides a barrier to transpirational water loss from the internal cells of the roots when the environment is dry (Benzing et al., 1982; Zotz and Winkler, 2013). Moreover, the presence of suberized cell layers at the periphery of the roots plays an important role in preventing water loss under condition of extreme drought (Hose et al., 2001). A thicker velamen radicum and suberized exodermis have also been observed in epiphytic orchids that grow in drier environment (Moreira et al., 2013). Therefore the presence of such structures in *C. corymbosa* may facilitate the maintenance of a suitable water balance for the entire plant, especially in dry seasons. By contrast, the lack of such structures in *P. albiflora* is connected with the phenology of this species, which precisely fits the timing of the Asian monsoon, i.e., producing roots at the beginning of the wet season and shedding them as the dry season approaches (Cribb and Butterfield, 1999). This phenomenon is also observed in species in other arid or semi-arid areas, such as the desert succulent *Agave deserti* (Hunt and Nobel, 1987). On account of the fact that hydraulics of root systems are more sensitive to drought than the leaves (Domec et al., 2009), and a few days without rainfall can be rather common even in the wet season (Zotz and Tyree, 1996; Engelbrecht et al., 2006), by not having to support a velamen radicum and suberized exodermis, plants of *P. albiflora* avoid over-investment in disposable roots as well as risk of dysfunction within the root hydraulic system that result from unexpected droughty periods occurring even during wet season in epiphytic habitats.

As an organ that stores water, carbohydrates and minerals, the pseudobulbs are of central importance to the growth and survival of epiphytic orchids (Ng and Hew, 2000). Similar to pseudobulbs in other orchids (Ng and Hew, 2000), those of our test species featured an impervious epidermis and multiple water-storing cells. However, those sampled pseudobulbs from *C. corymbosa* had significantly higher SWC values, indicating that this species has greater capacity to store water (Stratton et al., 2000). In addition, compared to the annual renewed pseudobulbs of *P. albiflora*, the pseudobulbs of *C. corymbosa* survive much longer than needed to support its foliage. In fact, in some epiphytic orchid species the pseudobulbs can survive as long as 8 years after their leaves dropped (Zotz, 1998). These old pseudobulbs can significantly increase the water storage capacity of a plant, thereby playing an essential role in helping species survive in prolonged periods of drought in epiphytic habitats, where availability of water is often severely limited (Zotz and Tyree, 1996; Zotz, 1999).

Leaf morphological traits play an important role in maintaining water balances in epiphytes (Zhang et al., 2015). Although phylogenetically related (Gravendeel et al., 2001) and sharing the same habitat, the two orchids examined here have contrasting leaf phenologies, leaf morphological traits, and leaf physiologies. The leaf lifespan of *P. albiflora* is 5–6 months under well-water conditions while plants of *C. corymbosa* can sustain their leaves for more than 4 years. These two species exhibit different trade-offs between CO_2_ acquisition and water loss. *C. corymbosa* invests more resources in the functions of water transport and reduction of water losses by constructing a thicker leaf epidermis and denser veins, whereas *P. albiflora* tends to fix more carbon during short, favorable growing seasons so that higher *A*_n_ and *g*_s_ values were found in *P. albiflora* under well-watered condition.

Leaf venation facilitates mechanical support, carbohydrate transport, and replacement of water lost via transpiration (Niklas, 1999; Roth-Nebelsick et al., 2001; Sack and Frole, 2006). The particular density of those veins can serve as a proxy for climate or habitat (Uhl and Mosbrugger, 1999; Zhang et al., 2015). Drought stress often leads to higher VD (Uhl and Mosbrugger, 1999; Dunbar-Co et al., 2009), which in turn influences the degree of plant drought resistance (Scoffoni et al., 2011). Therefore, the denser veins associated with *C. corymbosa* may help the evergreen leaves to survive the harsh dry season.

Although leaf cuticle is critical for protecting plants against water losses, its thickness is not always correlated to the cuticular water permeability (Riederer and Schreiber, 2001). Because leaf cuticle is more efficient in evergreen epiphytes than in deciduous epiphytes for preventing water losses after stomatal closure (Helbsing et al., 2000), we might speculate that other physical properties of the cuticle and the thickness of epidermis, rather than cuticle thickness, are responsible for the different *T*_70_ values for these two species.

Smaller and denser stomata enable plants to respond more quickly to environmental changes or to the decrease in leaf water potential, and they also promote greater diffusive conductance under favorable conditions (Aasamaa et al., 2001; Drake et al., 2013). These stomatal attributes are essential for the survival of *C. corymbosa* under conditions of fluctuating water availability. Although, by comparison, the large stomata of *P. albiflora* are slower to close and less able to prevent a hydraulic dysfunction in dry conditions, this lag in response may be advantageous in cool, moist, or shaded environments (Aasamaa et al., 2001; Hodgson et al., 2010) during the wet season.

Plants of *C. corymbosa* invested vast resources toward traits of different organs related to water supply and maintenance. These different strategies for resource investment affect their physiological performances under drought stress. More drought-resistant species control their stomatal functioning to allow for some carbon fixation at water stress, thus improving their WUE_i_ (Yordanov et al., 2000). Our study results suggest that the higher water storage capacity by the pseudobulbs of *C. corymbosa* facilitates this process, such that the leaf maintains a relatively higher water potential even when the water potential of the culture substrate becomes quite low after 25 days of imposed drought. The performances of these two orchids under stress are similar to those reported for co-occurring Mediterranean woody species. That is, plants with thin, high assimilation rate leaves are more vulnerable to drought while evergreen species, with thicker leaves and low assimilation rates, had the longest drought survival time (Lopez-Iglesias et al., 2014).

The genera *Pleione* and *Coelogyne* belong to the monophyletic subtribe Coelogyninae, and the deciduous *Pleione* diverged early to one of the three major clades (Gravendeel et al., 2001). In addition to their coexistence described here, other *Pleione* species have been recorded as co-existing with evergreen members in subtribe Coelogyninae, especially *Coelogyne* species (Cribb and Butterfield, 1999). Therefore, we speculate that a hydrological niche segregation (Silvertown et al., 2015) occurs between these two genera. The most common way for coexisting plants to achieve this type of segregation is by employing different strategies of water acquisition, such as through contrasts in their phenologies or rooting depths (Silvertown et al., 2015). For example, in seasonal tropical dry forests, evergreen trees can access soil water at greater depths than coexisting deciduous species during the dry season (Meinzer et al., 1999; Hasselquist et al., 2010). Because deep soil water is not available within an epiphytic habitat, epiphytes can survive the dry season only by using their stored water or by a very efficient water uptake during sporadic rainfall events (Zotz and Tyree, 1996). Previous researches in tropical ecosystems have predicted that increased drought would favor an increase in deciduous trees and a reduction in evergreen trees (Enquist and Enquist, 2011; Fauset et al., 2012). But these studies focused on annual precipitation rather than precipitation frequency, while the former is of lower relevance for epiphytic plants (Zotz and Bader, 2009). In contrast, our results suggest that evergreen epiphytes are better able to cope with drought events in the growing season. Thus, a change in precipitation frequency would have a larger impact on deciduous epiphytes than evergreen ones.

Zotz and Winter (1994) have shown that the annual carbon gain of three co-occurring epiphytic species is similar regardless their leaf phenology (evergreen vs. deciduous) or photosynthetic pathway (C_3_ vs. CAM). This indicates that the strategies of stress escape or stress avoidance in an epiphytic habitat can be equally beneficial in terms of annual leaf carbon gain. We found that 25-days of stress decreased the lifespan of leaves in *P. albiflora*, which inevitably led to the decrease of annual leaf carbon gain in this species. Therefore, the “winners” and “losers” of epiphytes after microclimate shifts (Padmawathe et al., 2004; Werner and Gradstein, 2008; Werner, 2011) probably reflect the different adaptive strategies utilized by epiphytic taxa in response to environmental disturbances.

## Conclusion

The two species investigated here differ significantly in their water-related traits, with plants of *C. corymbosa* having higher capacity for water uptake and storage and greater ability to reduce water losses. By comparison, plants of *P. albiflora* have higher photosynthetic capacity under well-watered conditions. The different photosynthetic performance responding to drought and re-watering in these two co-occurring epiphytic orchids is related to water-related traits. The divergence in morphology and physiology reflects fundamentally different adaptive strategies in the same epiphytic habitat. The drought escape plants are more vulnerable to the fluctuating water availability in an epiphytic habitat in the global climate change and anthropogenic disturbances. Therefore, more attention should be focused on such species in the conservation of epiphyte diversity in the changing world.

## Author Contributions

S-BZ and HH designed experiments. WZ carried out experiments and analyzed experimental results. WZ and S-BZ wrote the manuscript.

## Conflict of Interest Statement

The authors declare that the research was conducted in the absence of any commercial or financial relationships that could be construed as a potential conflict of interest.
